# Guidelines and quality measures for the diagnosis of optic ataxia

**DOI:** 10.3389/fnhum.2013.00324

**Published:** 2013-07-02

**Authors:** Svenja Borchers, Laura Müller, Matthis Synofzik, Marc Himmelbach

**Affiliations:** ^1^Division of Neuropsychology, Department of Cognitive Neurology, Centre for Neurology, Hertie-Institute for Clinical Brain Research, Eberhard Karls UniversityTübingen, Germany; ^2^Department of Psychiatry, Psychosomatics and Psychotherapy, University of WürzburgWürzburg, Germany; ^3^Department of Neurodegenerative Diseases, Hertie-Institute for Clinical Brain Research, Eberhard Karls UniversityTübingen, Germany; ^4^German Research Center for Neurodegenerative Diseases (DZNE), Eberhard Karls UniversityTübingen, Germany; ^5^Centre for Integrative Neuroscience, Eberhard Karls UniversityTübingen, Germany

**Keywords:** optic ataxia, cerebellar atrophy, cerebellar ataxia, cerebellum, parietal lobe, bedside test, reliability

## Abstract

Since the first description of a systematic mis-reaching by Bálint in 1909, a reasonable number of patients showing a similar phenomenology, later termed optic ataxia (OA), has been described. However, there is surprising inconsistency regarding the behavioral measures that are used to detect OA in experimental and clinical reports, if the respective measures are reported at all. A typical screening method that was presumably used by most researchers and clinicians, reaching for a target object in the peripheral visual space, has never been evaluated. We developed a set of instructions and evaluation criteria for the scoring of a semi-standardized version of this reaching task. We tested 36 healthy participants, a group of 52 acute and chronic stroke patients, and 24 patients suffering from cerebellar ataxia. We found a high interrater reliability and a moderate test-retest reliability comparable to other clinical instruments in the stroke sample. The calculation of cut-off thresholds based on healthy control and cerebellar patient data showed an unexpected high number of false positives in these samples due to individual outliers that made a considerable number of errors in peripheral reaching. This study provides first empirical data from large control and patient groups for a screening procedure that seems to be widely used but rarely explicitly reported and prepares the grounds for its use as a standard tool for the description of patients who are included in single case or group studies addressing optic ataxia similar to the use of neglect, extinction, or apraxia screening tools.

## Introduction

Optic ataxia (OA) was first described by Rudolph Bálint in 1909 as a neurological symptom resulting in gross mis-reaching to targets in the peripheral visual field (Balint, 1909). Initially, it was only seen as part of a triad of symptoms, which forms the basis of the Bálint-Holmes syndrome. Two additional symptoms are usually observed in a Bálint syndrome, namely oculomotor apraxia and simultanagnosia (Balint, 1909; Rizzo and Vecera, 2002). Garcin et al. (1967) were the first to report that OA can also appear as a distinct disorder in isolation. The defining characteristic of OA is the contrast between the occurrence of spatial errors in reaching movements to targets in the visual periphery and unimpaired movements to targets in the central visual field. This feature together with hand- and field-effects in unilateral cases supported the conclusion that OA represents a visuomotor coordination deficit that could neither be explained by a motor deficit nor by a sensory deficit alone (Perenin and Vighetto, 1988). In contrast to their visuomotor impairments, patients with OA seem to be able to detect and localize targets in their complete surroundings and reliably perceive shape, size and orientation of targets in the peripheral visual field (Perenin and Vighetto, 1988; but see Pisella et al., 2009). Performing a lesion analysis with sixteen OA patients, Karnath and Perenin (2005) found the lateral and medial parieto-occipital junction (POJ) to be specifically affected in OA.

Remarkably, the vast majority of the studies investigating OA patients report the behavior of only one or two patients (Jeannerod et al., 1994; Milner et al., 1999, 2001; Pisella et al., 2000; Roy et al., 2004; Himmelbach and Karnath, 2005; Blangero et al., 2007, 2008; Gaveau et al., 2008; Rice et al., 2008; Himmelbach et al., 2009; Jax et al., 2009; Cavina-Pratesi et al., 2010; McIntosh et al., 2011) with very few exceptions (Perenin and Vighetto, 1988; Blangero et al., 2010). Furthermore, many of the single-case reports examine the same patients repeatedly. Interestingly, although the same patients are tested, different authors come to different conclusions regarding the status of the patient. Patient CF is reported by Clavagnier et al. (2007) as a recovered patient: “[…] in the second patient (CF), the syndrome gradually improved after 3 months and eventually totally disappeared after 5 months.” (p. 25). In contrast, the same patient participated in multiple experimental studies over a period of at least 3 years as a chronic case of unilateral optic ataxia (Khan et al., 2005, 2009; Blangero et al., 2008; Granek et al., 2012). Whether such disagreements are due to variability in the patient's behavior, use of different diagnostical procedures, or a different interpretation of test outcomes remains unknown to us.

Case reports typically present a lot of clinical and experimental data due to an extensive investment of time and other resources for the examination of a single patient. Unfortunately, detailed information why and how the respective patient was recruited in the first place, i.e., the initial screening of the patient, is usually not reported. Detailed quantitative reports concerning movement kinematics from motion capturing systems etc. describe the respective behavior of a single patient in a particular experiment very precisely—e.g., the paradoxical improvement of delayed reaching (Milner et al., 1999, 2001, 2003; Himmelbach and Karnath, 2005). However, without a common agreement about a simple procedure that can be used in large patient populations rather than only in a few single cases, we do not know whether the occurrence of the clinical phenomenon OA is really linked to the observed experimental effects. Thus, it would be possible that there are several patients who would be diagnosed as OA cases without specific effects like the paradoxical improvement of delayed movements, questioning the postulated association between the phenomenology of OA and such experimental findings.

Optic ataxia seems to be of minor importance for the clinician. This very specific visuomotor deficit, causing troubles only for movements to targets in the retinotopic periphery, can be easily compensated for by looking at the respective targets before initiating a hand movement. Optic ataxia might become more important in a clinical context if it is combined with other disorders, e.g., as part of a Bálint-Holmes Syndrome (Rizzo and Vecera, 2002). The combination of optic ataxia with parietal impairments like oculomotor apraxia and/or simultanagnosia can result in severe limitations in everyday life because the patients are not able to fixate a target in the first place (Al-Khawaja and Haboubi, 2001; Gillen and Dutton, 2003; Rizzo and Vecera, 2002). Thus, the identification of optic ataxia in individual patients helps to render adapted neurorehabilitative strategies for those patients who suffer from debilitating combinations of parietal deficits (Al-Khawaja and Haboubi, 2001; Gillen and Dutton, 2003; Rizzo and Vecera, 2002). However, until today the impact of optic ataxia on everyday life in patients with multiple neuropsychological disorders has not been systematically investigated because this impairment is not well-known and because most clinicians do not know adequate behavioral tests. On the other hand, optic ataxia became more important in recent years being one of the core behavioral symptoms indicating the presence of a posterior cortical atrophy (PCA) (Mendez et al., 2002; Crutch et al., 2012). Because of a rather late onset of memory impairments in patients suffering from PCA, the onset of this dementing syndrome is often overlooked or misdiagnosed (Crutch et al., 2012). Interestingly, none of these studies mentioned how the presence of optic ataxia was detected in PCA patients.

Although other procedures, like tracing 2D figures as fast as possible (Kim et al., 2004), have been suggested, reaching for objects in the peripheral and central visual field in a way represents a standard test for optic ataxia since it was presumably tested in all of the reported patients at some time point. This screening procedure was described by Perenin and Vighetto (1988): “[…] the participant had to fixate the camera lens. He was asked to reach and grasp as quickly and accurately as possible an object (a big pencil) that was presented by the experimenter placed behind him at various locations successively in the ipsilesional hemifield, in the central field and then in the contralesional hemifield. First the hand ipsilateral and then contralateral to the lesion was tested. […] In condition 3, […] 1 object was presented but instead of fixating the camera lens the participant had to orientate eyes and head toward the object while reaching for it” (p. 652). The original report already included the data of a healthy control group. However, the size of this control group remained unclear. Based on the available information there were at least 5 but not more than 6 or 7 controls for the critical tasks. In contrast to experimental measurements with motion capturing systems, this screening can be conducted by every examiner and with most acute and chronic stroke patients. Unfortunately, so far there are no instructions or guidelines for the examination beyond the coarse description presented above. Furthermore, the reliability of this screening and the evaluation of the patients' performance has never been estimated.

Therefore, the aim of our study was to standardize and evaluate the bedside test for optic ataxia presented by Perenin and Vighetto (1988). We developed a set of instructions for the execution of the test and evaluation criteria for the scoring. First of all, we tested a large group of healthy participants to derive a cut-off score for the diagnosis of OA. Secondly, a large group of stroke patients was tested to assess the inter-rater reliability of the test and scoring procedure. Thirdly, the test was conducted a second time with a subgroup of patients to assess its test-retest reliability. Finally, to scrutinize the specificity of the test we examined a group of cerebellar ataxia patients who showed general motor coordination deficits. As OA is supposed to occur independently of such general coordination deficits a difference between peripheral and central visual field reaching is not expected in this patient group.

## Materials and methods

### Participants

Thirty-six neurologically healthy participants were tested in order to determine a cut-off score. All participants were right-handed and had normal or corrected-to-normal visual acuity. The same test was conducted with a group of 52 stroke patients who suffered an ischemia or hemorrhage. Patients were included in the study when they were able to sit up in bed and maintain visual fixation. Exclusion criteria were lesions limited to the brain stem, brain tumors, or metastasis and infectious or autoinflammatory diseases (e.g., encephalopathy, meningitis, vasculitis), known administration of narcoleptics (with motor disturbances as side effect) and additional brain disorders (e.g., Parkinson's disease, multiple sclerosis). Eleven of these stroke patients were chronic patients who suffered a stroke more than 390 days earlier (Table 1). We examined acute and chronic patients to increase the sample size for the interrater reliability analyses as much as possible. Twenty-eight of the stroke patients were tested twice in two consecutive sessions in order to assess the test-retest reliability. Again, we included 3 chronic patients to increase the sample size. Because of the obvious differences between the acute and chronic phase with respect to behavioral variability, we conducted a complementary test-retest analysis excluding the chronic cases. Finally, 24 patients diagnosed with cerebellar ataxia (CA) due to global cerebellar degeneration were recruited. All of these patients were examined by a specialist on degenerative ataxias (M.S.). None of them showed any clinical, electrophysiological or imaging evidence of extra-cerebellar disease involvement. Secondary causes of ataxia (e.g., ataxias of inflammatory, vascular or metabolic origin) were excluded in all patients by extensive serum and CSF analysis and MRI imaging. Out of the patients with genetically confirmed ataxia, only patients with Spinocerebellar Ataxia (SCA) type 6 were included as degeneration in this SCA subtype is known to be essentially limited to the cerebellum (Schöls et al., 2004). SCA 1, 2, 3, 7, and 17 were excluded, as these SCA subtypes are known to affect several non-cerebellar CNS structures. All demographic details and clinical data are given in Table 1. The experiment was conducted in accordance with the 1964 Declaration of Helsinki and all participants gave their informed consent prior to testing.

**Table 1:** **Demographic details for all participant groups**.

	**Healthy participants**	**Stroke patients inter-rater**	**Stroke patients test-retest**	**CA patients**
Number of participants	36	52	28	24
Mean age in years (range)	60.0 (43–73)	66.0 (28–85)	68.6 (50–85)	50.9 (13–80)
Gender	19 females	17 females	5 females	8 females
	17 males	35 males	23 males	16 males
HP	–	10 HP	1 HP	–
HA/QA	–	4 HA	4 QA	–
		8 QA		
Stereoptic vision (TNO)	32 intact	36 intact	22 intact	21 intact
Type of lesion	–	46 ischemia	25 ischemia	–
		5 hemorrhage	2 hemorrhage	
		1 both	1 both	
Side of lesion	–	24 left	15 left	–
		20 right	13 right	
		8 bilateral		
Time since onset	–	41 acute patients (mean 5.8 days, range: 1–40 days)	25 acute patients (mean 4.9 days, range: 1–20 days)	24 chronic patients (mean 2,967 days, range: 365–14,600 days)
		11 chronic patients (mean 1,542 days: 390–6,500 days)	3 chronic patients (mean 3,000 days, range: 700–6,500 days)	
SARA total score	–	–	–	mean 11.6 (range: 4–22)

*CA, Cerebellar ataxia; HP, hemiparesis; HA, hemianopia; QA, Quadrantanopia; Stereoscopic vision, rated as intact if the participants passed at least the first plate of the TNO; SARA, scale for the Assessment and Rating of Ataxia (Weyer et al., 2007)*.

### Procedure

**Figure 1:**
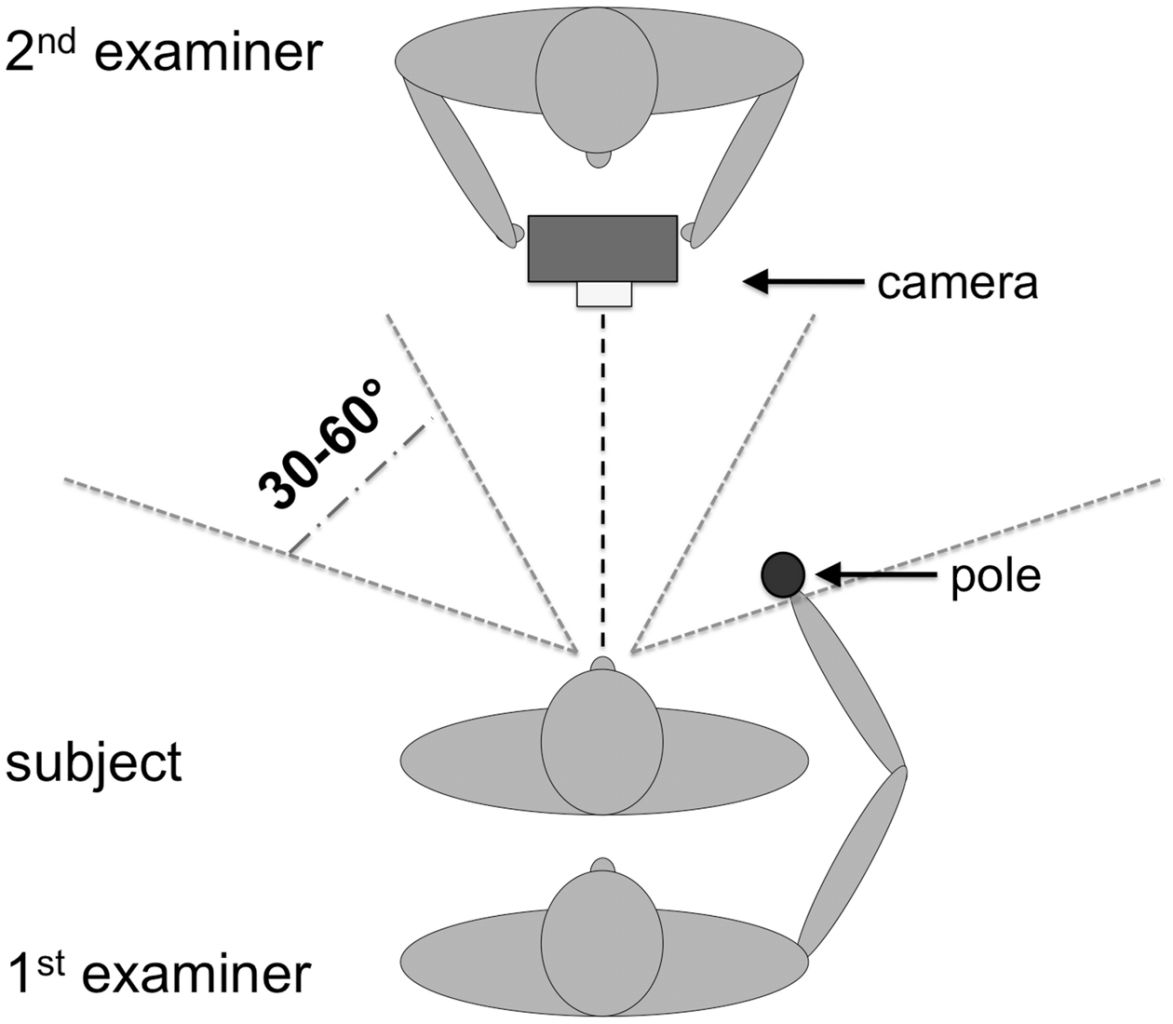
**Setup for the OA test procedure.** One examiner presented the pole to the participant while the other examiner observed the performance of the participants and controlled eye movements. The height for the target presentation varied between elbow height of the patient (with relaxed arms at the side of the body) and the top of his/her head.

**Figure 2:**
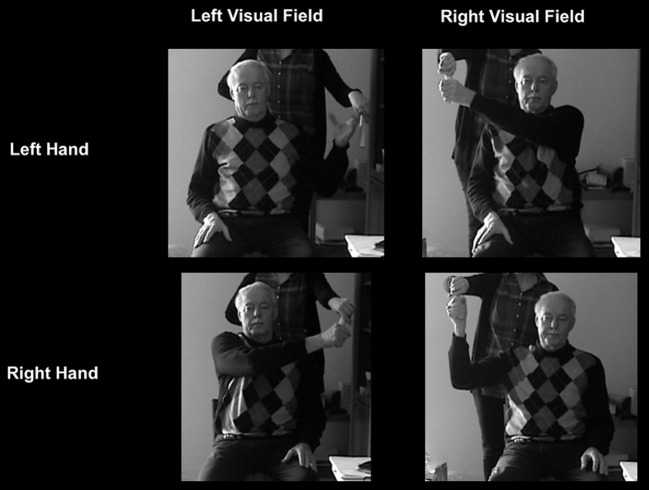
**OA patient with a right-hemisphere lesion grasps the pole in the different hand-field combinations during the fixation condition (peripheral grasping)**.

Before conducting the OA test a finger perimetry was conducted in order to assess visual field deficits such as hemianopia (HA) or quadrantanopia (QA). Secondly, the TNO test for stereoscopic vision (Laméris Ootech, www.ootech.nl) was conducted. Information about motor deficits such as a hemiparesis (HP) was derived from the routine clinical examination of each patient conducted by independent trained neurologists. Depending on the severity of the hemiparesis, the test for OA was conducted only with the non-affected hand. All tests were administered at the patients' bedside (with the patient sitting on the edge of the bed or with highly upright backrest in bed) or with the patient sitting on a chair in the patient's room. During the OA test, the participants' upper body was upright so that both arms could be moved freely. Each participant was tested in two conditions. In the first condition, he/she was instructed to look straight ahead (fixation), while in the second condition, the participant was instructed to look directly at the target object (saccade) while grasping. The examiner stood behind the participant and held a 20 cm long wooden pole (8 cm for the examiner to hold the pole and 12 cm for the patient to grasp) with a diameter of 2.8 cm to the side of the participants' body. The position of the pole was varied in a confined space: it was presented at a height between the participant's elbow and head, with a horizontal rotation angle around the body midline between 30 and 60° to the left and right, respectively, and the participant's arm was not completely extended for the maximum distance of the target object to the body (Figure 1). The pole was always presented in an upright orientation. The examiner varied the delays between individual trials to prevent anticipatory responses. The pole was presented in both visual hemifields, and the participant grasped it in each hemifield with the left and the right hand, respectively, resulting in four different hand/field combinations per condition (Figure 2). In each hand/field combination 10 movements were executed. Thus, in total 80 trials were performed. However, if fixation was not maintained the respective trials were repeated and the number of trials increased accordingly. Furthermore, the participant was instructed to grasp the pole along its visible extent with the full hand using a power grip. After each trial the participant moved his/her hand back to the resting position at the thigh. With a subgroup of stroke patients, the OA test was conducted twice. The time between the testing sessions was between one and 4 days (mean: 1.39, *SD*: 0.8). The OA test was videotaped by a second examiner in front of the patient, who also controlled fixation of the patients' gaze during the test sessions (Figure 1).

### Data analysis

Based on prior experience and inspections of the first video recordings a graded rating scheme for movement errors was developed (Table 2). This rating scheme associates different error types, ranging from minor spatial errors and hesitant movements to gross spatial misreaching, with ordinal categories between 0 (no errors) and 3 (gross errors). The categories 2 and 3 are similar to the notion of “corrected” and “uncorrected” errors in the report of Perenin and Vighetto (1988). All video recordings of patient examinations were then processed independently by two examiners (Svenja Borchers and Laura Müller) using this rating scheme.

**Table 2:** **Guidelines for scoring the performance on the OA test**.

**Error points**	**Description**
0 points	Participant grasps pole in one fluent movement
1 point	Participant grasps pole in one fluent movement at the lower edge only (at most three fingers touch the pole whereas the others fail) Participant touches the pole only with the finger tips and completes full hand grip in a second movement Participant falters or hesitates during hand transport to the target but eventually reaches the pole
2 points	Participant does neither grasp nor touch the pole with any finger in the first movement but grasps it in a second movement after an almost complete stop in between Participant jolts the pole during the first movement (e.g. with the back of the hand) but correctly grasps it in a second move
3 points	Participant does neither grasp the target in the first nor in any following movement

After rating all trials of a participant, the scores for each hand/field combination were summed up per visual condition, resulting in eight summed error scores. The actual number of valid trials per condition finally varied between 9 and 11. Therefore, each of the eight error scores was transformed into a percentage score relative to the possible maximum score: error score/(number of trials × 3) × 100. A difference value was calculated by subtracting the percentage error score of the fixation condition of one hand/field combination from the percentage error score of the saccade condition of the same hand/field combination. Factors that are supposed to influence both conditions, such as mild hemiparesis or tremor should thus be cancelled out. Furthermore, the difference score could also control individual differences in the rating of the observed movements as the personal interpretation of individual raters, influences the scores in both conditions. For all following analyses, we used the difference value of the percentage error score.

All statistical analyses were performed with SPSS (Version 19.0; SPSS, Inc. Chicago, IL, USA). Cut-off scores were determined in order to differentiate between normal and pathological test performance based on the performance of healthy participants for each hand/field combination. Two standard deviations were added to the mean of the controls' data (2 *SD*): Cut-off_(hand X, field Y)_ = Mean_(hand X, field Y)_ + [2 × Standard Deviation_(hand X, field Y)_]. This method is based on the fact that, under the assumption of a normal distribution of the controls' data and assuming that the control sample's values are identical with the parameters in the general population, 95.45% of the values lie within two standard deviations of the mean. Another method to compare single patients with the performance of a group of healthy participants is the use of adjusted t-statistics as suggested by Crawford and Garthwaite (2005). Using their program (SINGLIMS), we calculated two cut-off values corresponding to error probability thresholds of *p* < 0.05 (C&G 0.05) and *p* < 0.01 (C&G 0.01).

In order to determine whether the cut-off scores effectively differentiate normal from abnormal test performances, the test performance of the CA patients was evaluated, since CA patients were not necessarily expected to show OA symptoms.

Two different kinds of reliabilities were assessed for the scores of the OA test. On the one hand, the inter-rater reliability was calculated as the correlation of both experimenters' ratings for each hand/field combination across all stroke patients. We calculated the Pearson's correlation coefficient for parametric scores. Furthermore, we calculated the test-retest reliability, the Pearson correlation between the scores produced by one experimenter (Laura Müller) for two test sessions taking place on two different days.

## Results

The performance of the healthy control participants for each hand/field combination was assessed. An ANOVA between the difference values of the percentage error scores of the different hands and fields revealed a significant hand × field interaction [*F*_(1, 35)_ = 19.184; *p* < 0.001]. In particular, participants produced more errors in the left visual field with their left hand than with their right hand [*t*_(35)_ = −3.0; *p* = 0.005], while in the right visual field, more errors were made with the right hand than with the left one [*t*_(35)_ = 4.012; *p* < 0.001]. The performance was thus better in the incongruent (RH-LF, LH-RF) than in the congruent conditions (RH-RF, LH-LF) [*t*_(35)_ < −3.39; *p* < 0.003]. The impairment in OA patients should primarily differ between the combinations contralesional hand/contralesional field and ipsilesional hand/ipsilesional field, i.e., in congruent conditions. Thus, in the following we focused on the mean error scores across congruent hand/field conditions in healthy controls and cerebellar patients and the contralesional hand/contralesional field in stroke patients. If patients suffered bilateral lesions we included the maximum error score of the two congruent hand/field conditions in our analyses.

### Distribution of error scores

**Figure 3:**
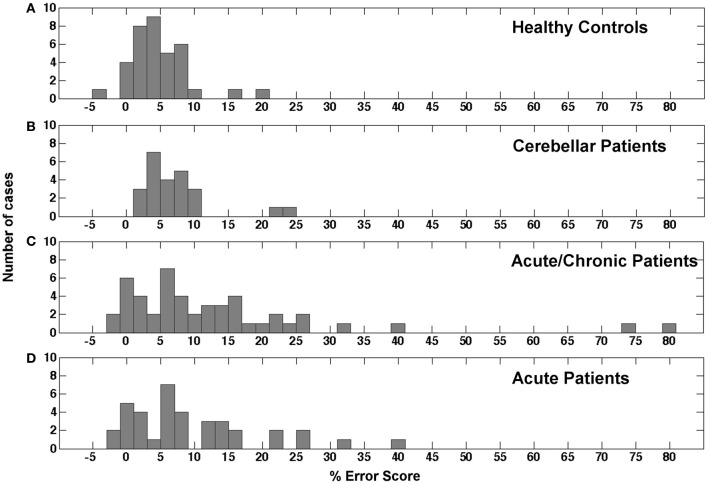
**Histogram showing the distribution of error scores in the study's different samples. (A)** Mean error scores for both congruent conditions in healthy controls. **(B)** Mean error scores for both congruent conditions in cerebellar patients. **(C)** Error scores for the contralesional hand and contralesional field condition in acute and chronic stroke patients. **(D)** Error scores for the contralesional hand and contralesional field condition in acute stroke patients only.

The distribution of error scores resulting from a comparison between reaching in the peripheral and reaching in the central visual field in different populations is currently unknown. As mentioned above only Perenin and Vighetto (1988) reported the outcome of peripheral reaching for a small healthy control sample of unknown size and a selected group of parietal patients. Figure 3A reports the distribution of mean difference scores for the congruent hand/field conditions in our sample of healthy controls. With a mean of 4.74% and a *SD* of 4.44% the values seemed to be roughly normally distributed with two outliers above 15%. Including the two outliers the distribution showed a positive skewness of 1.226 (*SE* = 0.393) and a kurtosis of 2.945 (*SE* = 0.768). A Kolmogorov-Smirnov test showed a significant deviation from a normal distribution [*D*_(36)_ = 0.150, *p* = 0.038]. Excluding these outliers resulted in a mean of 3.98% with a *SD* of 3.18%. The skewness changed to −0.126 (*SE* = 0.403) and the kurtosis to 0.383 (*SE* = 0.788). Without the two outliers no deviations from a normal distribution were detected by the Kolmogorov-Smirnoff test [*D*_(34)_ = 0.107, *p* = 0.200]. Figure 3B shows the distribution for the congruent hand/field conditions in the group of cerebellar patients. Again, the values seemed to be roughly normally distributed with a mean of 7.16% and a *SD* of 5.59% and two outliers above 20%. Including the two outliers the distribution showed a positive skewness of 2.02 (*SE* = 0.472) and a kurtosis of 4.333 (*SE* = 0.918). A Kolmogorov-Smirnov test showed a significant deviation from a normal distribution [*D*_(24)_ = 0.188, *p* = 0.028]. The exclusion of two outliers resulted in a mean of 5.71% with a *SD* of 2.78%. The skewness changed to 0.165 (*SE* = 0.491) and the kurtosis to −0.829 (*SE* = 0.953). Without the two outliers no deviations from a normal distribution were detected by the Kolmogorov-Smirnoff test [*D*_(22)_ = 0.112, *p* = 0.200]. The distribution of error scores for the contralesional hand/contralesional field of 48 acute and chronic patients is shown in Figure 3C. Four patients were not included because error scores could not be derived for the respective contralesional hand/field either because of a severe hemiparesis or hemianopia. In contrast to the distribution of healthy controls and cerebellar patients, the distribution of the stroke patients' scores showed a larger variability with a mean of 13.06% and a *SD* of 16.35%. The skewness of this distribution was 2.713 (*SE* = 0.343) and the kurtosis was 8.731 (*SE* = 0.674). The Kolmogorov-Smirnoff test showed a highly significant deviation from a normal distribution [*D*_(48)_ = 0.194, *p* < 0.001]. Figure 3D shows the distribution of the stroke patients' scores after the exclusion of the chronic patients. Still, the distribution of the stroke patients' scores showed a high variability with a mean of 9.64% and a *SD* of 9.76%. The skewness of this distribution was 1.282 (*SE* = 0.0.388) and the kurtosis was 1.428 (*SE* = 0.759). Again, the Kolmogorov-Smirnoff test showed a highly significant deviation from a normal distribution [*D*_(37)_ = 0.201, *p* = 0.001].

In conclusion, the error scores of healthy controls and cerebellar patients were similarly distributed. Both groups showed a roughly normal distribution with a small number of outliers that were in both groups more than 2 *SD* away from the respective group's mean value. The distribution of error scores in the stroke patients showed a higher variability and a longer tail in the positive range with and without the values of chronic patients.

### Cut-off scores

To provide a first guidance to other users of this screening method and our normalization data and to further inspect the data of the stroke patient sample we calculated a set of possible cut-off values based on accepted and statistically valid methods with reference to the distributions of healthy controls' values and the values from the cerebellar patients. This calculation also gives an impression of how many cases are likely to be detected by this screening method in an unselected sample of patients with an acute cerebral stroke. A first cut-off was calculated using the standard method (mean + 2 standard deviations) based on the healthy control's values resulting in a score of 14% in the congruent conditions (Table 3). Using this cut-off, 7 of 37 consecutively admitted acute patients would be detected with a pathological difference between peripheral and central reaching. Calculating a cut-off value by means of the adjusted *t*-test for single-case statistics suggested by Crawford and Garthwaite (2005) resulted in an error score of 13% for a threshold of *p* < 0.05 and an error score of 16% for a threshold of *p* < 0.01 (Table 3). Using the threshold of *p* < 0.05 resulted in 11 positive cases out of 37 examined acute patients, while the threshold of *p* < 0.01 identified 7 cases. Cut-off values derived from the cerebellar patients (Table 3) resulted in 6 detections for the standard method, 6 detections for C&G 0.05, and 2 detections for C&G 0.01. For both sets of cut-off values we included the respective outlier cases mentioned above. Excluding the small number of outliers would reduce the respective threshold values and result in a higher number of positive cases. Means and standard deviations for both control samples of our study without outliers are reported above in the results section. Thus, every researcher and clinician can derive additional thresholds using methods based on parametric inferential statistics or simply decide to use the maximum values in healthy controls or cerebellar patients that we observed here as a threshold for a clinical decision.

**Table 3:** **Cut-off scores**.

	**RH-LF**	**RH-RF**	**LH-RF**	**LH-LF**	**Congruent conditions**
**HEALTHY CONTROLS**					
Mean (*SD*)	2.48 (3.83)	4.53 (4.24)	1.54 (2.04)	4.95 (5.86)	4.74 (4.45)
Cut-off 2 *SD*	10.2	13.0	5.6	16.7	13.6
Cut-off C&G 0.05	9.1	11.8	5.1	15.0	12.4
Cut-off C&G 0.01	12.0	15.1	6.6	19.5	15.8
**CEREBELLAR PATIENTS**					
Mean (*SD*)	3.40 (4.57)	8.31 (8.97)	2.94 (3.21)	6.01 (4.19)	7.16 (5.47)
Cut-off (2 *SD*)	12.5	26.3	9.4	14.4	16.8
Cut-off (C&G 0.05)	11.4	24.0	8.6	13.4	21.2
Cut-off (C&G 0.01)	15.1	31.2	11.2	16.7	26.7

*The mean percentage error scores are given with their standard deviations for all hand-field combinations (RH, right hand; LH, left hand, RF, right visual field, LF, left visual field) and for the congruent conditions together (RH-RF and LH-LF). The resulting cut-off scores (rounded) are given in parentheses*.

### Specificity

The cerebellar patients suffered from motor coordination deficits (dysmetria and/or tremor) due to global cerebellar degeneration. None of these patients showed evidence of brain damage to the parietal cortex on routine MRI. The actual performance of these patients in the clinical OA screening thus should reveal the specificity of the OA screening in differentiating between the performance of patients with OA and patients suffering from another motor coordination deficit. The distribution of the error scores shown in Figure 3 showed that the range and distribution of values was very similar between the healthy controls and the cerebellar patients. We further examined the difference scores for each congruent hand/field combination (RHRF, LHLF) of each cerebellar patient individually against the cutoff values derived from the healthy controls sample (Table 4). With these comparison, which were equivalent to the single case analyses of the acute stroke patients, three out of 24 CA patients showed test scores higher than the healthy controls' cut-off values of 14% (2 *SD*) and 16% (C&G 0.01), or four patients based on the 13% cut-off (C&G 0.05).

**Table 4:** **Difference values of the percentage error scores of CA patients for congruent conditions**.

**Patient number**	**RH-RF**	**LH-LF**
1	30.77*	15.15
2	0.00	3.33
3	0.00	3.03
4	10.42	10.53
5	11.11	10.00
6	5.56	5.56
7	5.13	5.56
8	1.75	12.28
9	11.76	5.77
10	0.00	2.56
11	3.03	3.33
12	5.56	10.00
13	3.03	6.67
14	35.56*	11.04
15	5.83	2.73
16	19.44*	−3.03
17	13.33	0.51
18	2.78	8.33
19	7.78	0.00
20	11.11	3.33
21	2.38	7.02
22	3.03	4.76
23	0.00	6.67
24	10.00	9.09

*Error scores above the C&G 0.01 cut-off are marked with an asterisk*.

### Inter-rater reliability

**Figure 4:**
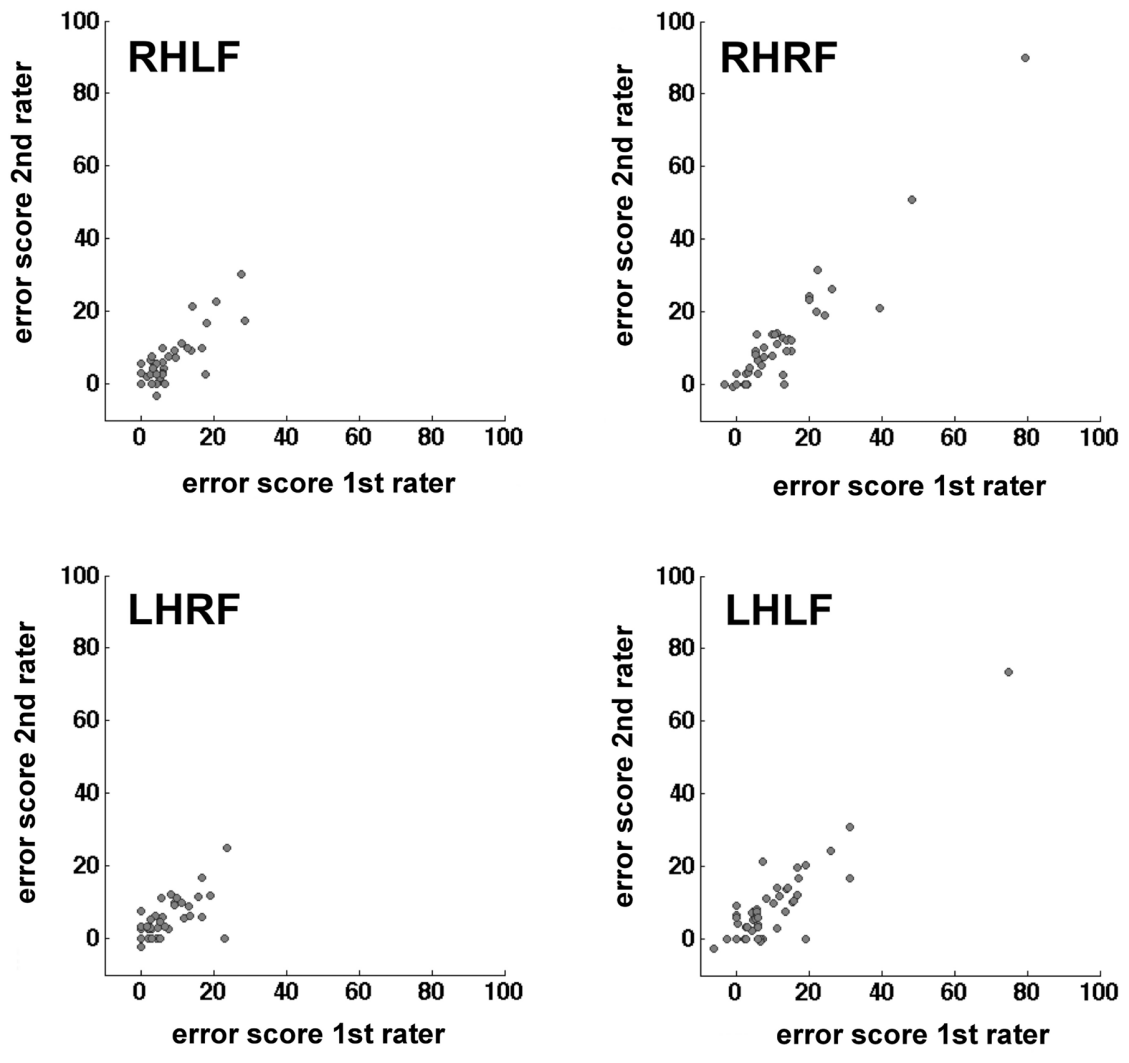
**Scatter plots mapping the correlation of the two raters per hand/field combination.** Error scores are differences between percentage error scores in the fixation and saccade condition.

The calculation of the inter-rater reliability was based on the complete stroke patient data set of 52 patients. The correlation between the results of the two raters was calculated for the difference value of each hand/field combination, respectively. There was a small variability of the number of participants for the different hand/field combinations due to a drop-out owing to HA or HP affecting only a specific visual field or hand. The correlations for all hand/field combinations ranged between *r* = 0.718 and *r* = 0.947 (Table 5). The scatter plots of the error scores displayed extreme values in two of the four conditions (Figure 4). Even when those three outliers (>mean + 3 *SD*'s) were removed, the resulting correlations ranged between *r* = 0.718 and *r* = 0.849 (Table 5).

**Table 5:** **Results for the inter-rater reliability**.

**Condition**	**Full data set**			**Excluding extreme values**		
	**No. of participants**	***r***	***p***	**No. of participants**	***r***	***p***
RHLF	47	0.843	<0.001	47	0.843	<0.001
RHRF	48	0.947	<0.001	46	0.849	<0.001
LHRF	50	0.718	<0.001	50	0.718	<0.001
LHLF	50	0.899	<0.001	49	0.750	<0.001

*Pearson correlation coefficients (r) were calculated between the difference values of the percentage error scores evaluated by two raters independently for all hand-field combinations (RH, right hand; LH, left hand, RF, right visual field; LF, left visual field)*.

### Test-retest reliability

**Figure 5:**
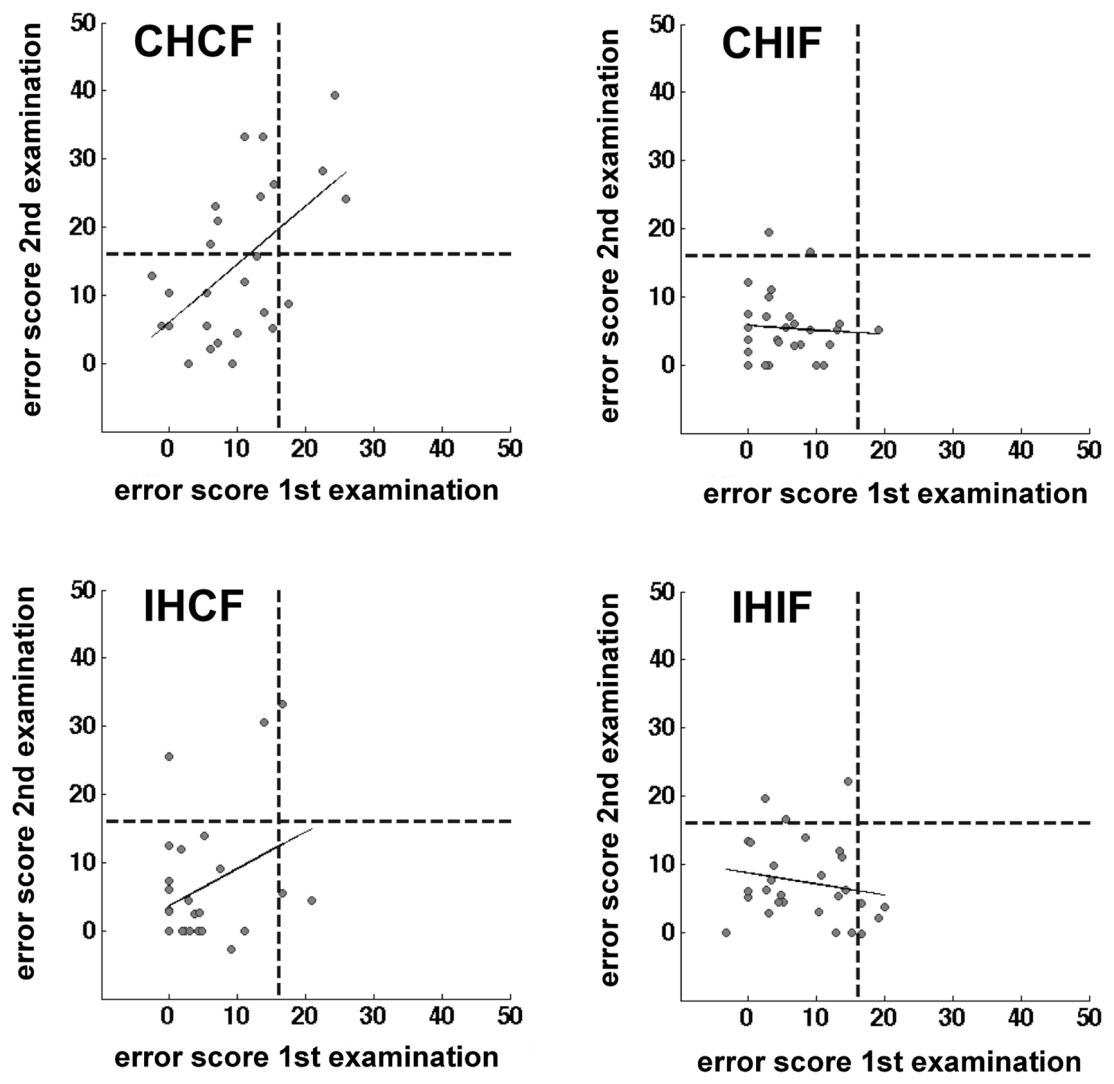
**Scatter plots showing the correlation of two test sessions per hand/field combination.** Error scores are differences between percentage error scores in the fixation and saccade condition The C&G 0.01 cut-off value is indicated by the black dotted lines. The correlation is indicated by the black line.

We assumed that, without any pathological process, most of the day-to-day variability in the healthy controls would be driven by random processes. The OA screening is definitely not sensitive enough to measure the subtle differences in the sensorimotor capabilities of healthy humans. Therefore, we used only patient data for the test-retest reliability analysis. The error scores of 28 unilateral acute and chronic stroke patients who were tested in two separate sessions on two different days were reorganized with respect to lesion side. We found a significant correlation only for the contralesional field and hand combination (*r* = 0.578, *p* = 0.001). In contrast, no significant correlations could be identified in the other combinations (Table 6 and Figure 5). The ipsilesional hand/contralesional field combination also resulted in a positive correlation (*r* = 0.348). Obviously, day-to-day variability might be different in acute and chronic patients. In agreement with this assumption the correlation calculated only for the group of acute patients was slightly lower for the contralesional hand/field combination (*r* = 0.551, *p* = 0.004) (Table 6).

**Table 6:** **Results for the test-retest reliability**.

**Condition**	**Acute and chronic patients**			**Excluding chronic patients**		
	**No. of patients**	***r***	***p***	**No. of patients**	***r***	***p***
CHCF	28	0.578	0.001	25	0.551	0.004
CHIF	28	−0.067	0.735	25	−0.057	0.787
IHCF	28	0.348	0.070	25	0.365	0.073
IHIF	28	−0.180	0.360	25	−0.158	0.450

*Pearson correlation (r^2^) was calculated between the difference values of the percentage error scores of two test sessions for all hand-field combinations (CH, contralesional hand; IH, ipsilesional hand; CF, contralesional visual field; IF, ipsilesional visual field)*.

Interestingly, only three cases showed values above the threshold in both test sessions with the C&G 0.01 cut-off value (16%) with 17 cases being consistently below the threshold in both sessions. One patient was above the threshold in the first test but not in the second, seven other patients showed the reverse pattern (Cohen's kappa = 0.282, *p* = 0.077). This pattern changed somewhat with the C&G 0.05 cut-off (13%). Five patients were repeatedly identified as optic ataxic cases, 14 cases were consistently negative, three patients were pathological only in the first testing session, and five patients only in the second session (Cohen's kappa = 0.381, *p* = 0.041). The standard cut-off (14%) resulted in four consistently positive detections, 15 consistent negative results, two cases with a positive result in the first testing session only, and seven patients with a positive result in the second testing session only (Cohen's kappa = 0.267, *p* = 0.121). Excluding the three chronic patients also changed the results for the interrater agreement, again we found a significant agreement between test-retest decisions based on the most liberal Crawford and Garthwaite threshold (C&G 0.01: Cohen's kappa = 0.187, *p* = 0.238; C&G 0.05: Cohen's kappa = 0.386, *p* = 0.045; 2 *SD*: Cohen's kappa = 0.259, *p* = 0.119).

## Discussion

The purpose of this study was to evaluate, standardize, and communicate a bedside screening procedure for the diagnosis of OA that was already introduced by Perenin and Vighetto (1988). To investigate its usability and reliability and the typical range of error scores we examined a large group of healthy controls, a large cohort of stroke patients with cerebral lesions, and a group of patients suffering from cerebellar atrophy. We derived cut-off values based on established statistical methods from a neurologically healthy control group and a group of cerebellar patients and further examined the specificity of this screening procedure. Our study provides an empirical basis for the use of this procedure in different places by different examiners demonstrating that, under consideration of a few recommendations and standards communicated with this report, the test outcome is reliable between different raters and as reliable as other clinical screening instruments in repeated examinations.

We found a rather high correlation between two independent raters when using the proposed guidelines for the analysis of the observed behavior. Our results are comparable with the results of other studies assessing the correlation of two or more raters for clinical instruments and procedures. Vanbellingen et al. (2010) investigated the inter-rater reliability of a diagnostic instrument for the detection of upper limb apraxia. They reported correlation coefficients ranging from 0.65 to 0.99. South et al. (2001) evaluated the inter-rater reliability of three published scoring systems for the clock drawing task and reported correlation coefficients between 0.51 and 0.95. A study conducted by Coderre et al. (2010) assessed the sensorimotor function of stroke patients with a visually guided reaching task for which they reported an inter-rater reliability between 0.77 and 0.97. Therefore, the inter-rater reliabilities of the present study ranging between 0.72 and 0.95 are comparable with other clinically relevant instruments and procedures.

For the test-retest reliability, we found only a moderate correlation of 0.578. However, mostly acute patients, 25 of 28, were tested to assess the test-retest reliability, most of them within seven days post-stroke. In this acute stage a patient's performance might change within days or even hours. Such spontaneous recovery, or deterioration of health, is an important factor influencing the reproducibility of test scores. For the test to be useful as a screening tool for acute patients we recommend to repeat the test at least twice in order to reduce the number of false positives and false negatives. However, this result for the optic ataxia screening was neither surprising nor particularly poor. Other methodological studies of clinical screening instruments reported similar test-retest reliabilities. Diederich and Merten (2009) evaluated the test-retest reliability of the bicycle drawing test. They found a correlation of 0.58 for an interval of only 1 day between the first and second testing and a correlation of 0.73 for an interval of 3 days between testing (Diederich and Merten, 2009). Dikmen et al. (1999) examined test-retest reliabilities for a broad range of neuropsychological measures. They found that while most measures (e.g., Boston Naming Test, Stroop Test, Name Writing) resulted in correlations between 0.66 and 0.92, some widely used memory tests showed much lower correlations. The established and highly standardized Wechsler Memory Scale showed a test-retest reliability between 0.58 and 0.83 and the Selective Reminding test showed correlations between 0.46 and 0.64 (Dikmen et al., 1999). Thus, other clinically important instruments show test-retest reliabilities in a similar or even lower range as the correlation that we found for the optic ataxia screening here. Obviously, further investigations are needed to determine the test-retest reliability including more cases with a clear-cut optic ataxia. Due to the small number of such patients this would either require a much longer data acquisition period and/or the combined efforts of a multicenter study.

Calculating explicit threshold values we did not intend to provide a gold standard for the diagnosis of optic ataxia. We calculated six cut-off scores based on the performance of a healthy control sample and a sample of cerebellar patients, comparing the performance for extrafoveate and foveated targets. Interestingly, in healthy controls we observed less errors in the incongruent conditions, i.e., when grasping with the left hand to the right visual field or vice versa. This combination of start position and target location allowed to grasp the target object successfully with less precise, sweeping movements. OA patients, however, should have most difficulties when reaching to a target with their contralesional hand in their contralesional visual field. As the primary objective of this test procedure and the use of cut-off scores is the detection of possible OA cases, we only used the congruent conditions in the healthy control group for the calculation of cut-off scores. The mean error scores for the congruent conditions in healthy participants in our measurements were very close to the mean error scores reported by Perenin and Vighetto (1988) for their small control group. These authors reported mean error values for reaching to a visual object in the peripheral field without saccades of about 12% for each condition. This percentage was based on the number of error trials related to the total number of trials. Perenin and Vighetto (1988) stated that, in healthy controls, they observed only corrected trials. This means, based on an average of 10 trials per person and condition that most controls showed only one corrected error trial. Applying our rating scheme to the behavioral outcome in controls from Perenin and Vighetto results in a percentage error score of 6.7%. Taking into account that the numbers reported by Perenin and Vighetto did not consider any smaller errors for the saccade-condition and their much smaller sample size of a different age range, this estimated value of 6.7% based on their report is very close to the average of 4.74% from the congruent conditions in our healthy control sample. However, using the thresholds calculated here from the healthy controls resulted in the detection of a rather high number of positive cases among the acute stroke patients up to 11 with the most liberal, but statistically sound threshold. This might be due to general differences between a hospitalized patient sample on the one hand and a control sample of volunteering healthy controls on the other hand. Thus, we additionally calculated thresholds based on the sample of cerebellar patients resulting in six positive cases for the more liberal thresholds, but only two for the most conservative threshold of an error score of 26.7%. The latter threshold detected only cases that showed error scores higher than any control despite of two cerebellar patients. This result of our investigations into statistically sound threshold values shows that the small healthy control group of Perenin and Vighetto (1988) conveyed a wrong impression. Healthy controls and neurological patients with error scores higher than 4–7%, i.e., more than one corrected error trial, are more common than previously assumed. Our suggestion regarding the use of cut-off values is rather pragmatic. If the recruitment of patients for experimental studies, investigating what specific effects are associated with the presence of optic ataxia and which are not, motivates the clinical screening, the number of false positives in a first screening would be less important and the lower C&G 0.05 threshold (13%) would be a good choice. If, on the other hand, the number of false positives is particularly important, we would suggest to adopt the most conservative threshold based on the sample of cerebellar patients of 26.7%. Alternatively, one might use the highest differential score that we observed in a cerebellar control for one hand/field combination of 36% (Table 4). Our report of mean values and standard deviations from both control populations with and without outliers and the respective outcome of our examinations in a large stroke sample allows other researchers and clinicians to reach their own conclusions on practical thresholds for their research and clinical work.

Obviously, it is not clear whether any of the cerebral patients who scored above the respective thresholds in our study really showed true optic ataxia. We examined the visual field of these patients only with a finger perimetry. We would not have detected any more subtle visual problems like reduced contrast sensitivity or blurred vision. Also higher order visual deficits, like impairments in size, orientation, or location discrimination (Pisella et al., 2009), would have gone unnoticed in this screening. Furthermore, we did not test for proprioceptive deficits in detail. In conclusion, we do not know whether anyone of these patients really suffered from true optic ataxia in agreement with the negative definition that excludes any purely sensory or motor disorders (Perenin and Vighetto, 1988). Yet, it was not the intention of our study to report the incidence of ‘true’ optic ataxia patients but to investigate and communicate the usability of a screening procedure, its reliability, and the range of typical results in a healthy control sample, a patient control sample, and an unselected group of consecutively admitted stroke patients. In doing so we hopefully motivate others to use this procedure to provide additional information on a patient's status for clinical and experimental reports of single cases and groups of patients where the presence of optic ataxia might be an issue to be considered, comparable to the use of simple screening tests for other neuropsychological disorders, e.g., for apraxia (Goldenberg, 1996).

In conclusion, the data reported here indicate that the reported guidelines for conducting the test and evaluating the test results are applicable as a screening instrument for diagnosing OA in a clinical setting. We have shown that two independent raters produced very similar scores using the described guidelines. We provided an estimate for the test-retest reliability and showed that test results were moderately reproducible on different days even in acute patients, comparable to other clinical instruments that are widely used in clinical practise. Our study reports the expected range of values for participants who did not experience a cerebral brain damage and, most certainly, did not suffer from optic ataxia. Thus, our work provided first empirical data from larger groups for a screening procedure that seems to be widely used but rarely explicitly reported and prepared the grounds for the use of this screening procedure as a standard tool for the description of patients who are included in single case or group studies similar to the use of neglect, extinction, or apraxia screening tools. Such a common use of this screening, keeping in mind also its limitations shown here, would allow for more conclusive comparisons across different studies and clinical reports.

### Conflict of interest statement

The authors declare that the research was conducted in the absence of any commercial or financial relationships that could be construed as a potential conflict of interest.

## Appendix

### Instructions for conducting the optic ataxia bedside test

#### Test procedure

The participants' upper body should be upright so that both arms can be moved freely. Ideally, the participant should sit on a chair. If this is not possible the participant should either sit on the edge of the bed or with highly upright backrest in bed. One examiner stands in some distance to the participant and controls fixation. The other examiner stands behind the participant and holds a wooden pole to the side of the participants' body. The test is conducted in two conditions. In the first condition the participant is instructed to look straight ahead (fixation) while in the second condition the participant is instructed to look directly at the pole while grasping. The position of the pole is altered on three axes: height (between elbow and head), distance to the midline (between 30 and 60°) and distance to the body (the participants' arm should not be completely extended). The examiner varies the rhythm in which the pole appears in order to prevent anticipatory responses. The pole is presented in both visual fields and within each field the patient grasps the pole either with the left or with the right hand. In each hand/field combination 10 grasping movements are executed. If fixation is not maintained the number of trials should be increased accordingly. The participant is instructed to grasp the pole with the entire hand in a way that all fingers touch the pole. After each trial the grasping hand should be laid down on the leg in order to guarantee a complete grasping movement each time. If the patient does not move back the hand completely the number of trials should be increased accordingly.

### Participant instructions

I am going to hold this pole at the left and right side of your body. You are supposed to grasp the pole with the whole hand in one fluent and uninterrupted movement. I will always indicate the hand that should be used for grasping. After each grasping movement you should place your hand on your leg and start the following movement from there. Start grasping not until you actually see the pole on the side of your body.

***Condition 1: Fixation***. To start with, you should look straight into the camera and not directly to the pole during the whole grasping condition. I will position the pole first at the right/left side of your body and as soon as you see it you should grasp it with your left/right hand.

***Condition 2: Saccades***. Now we will repeat the procedure, but this time you do not have to look straight ahead anymore. Instead, you should look directly at the pole. Only start the grasping movement when you can actually see the pole. I will again position the pole first at the right/left side of your body and as soon as you see it you should grasp it with the left/right hand.
